# Calcium hydroxide recycled from waste eggshell resources for the effective recovery of fluoride from wastewater†

**DOI:** 10.1039/d2ra05209a

**Published:** 2022-10-04

**Authors:** Wenjing Chen, Yuanyue Wu, Zhiyin Xie, Yiyuan Li, Weitai Tang, Jinbei Yu

**Affiliations:** College of Resources and Environment, Chengdu University of Information Technology No.24 Xuefu Road, Shuangliu District Chengdu 610225 China wenjingchen88@126.com +86 28 85966913 +86 28 85966913

## Abstract

In the hunt of waste recovery pathways, eggshells emerged as a potential adsorbent for fluoride because they contain plenty of calcium. However, as the main component, calcite has weak interaction with fluoride. In this study, calcium hydroxide was derived from waste eggshells successfully by an aging treatment with moisture for fluoride recovery from water. The X-ray diffraction (XRD) and infrared spectroscopy (FT-IR) analyses indicate that CaO in calcined egg shells (AEG900) is completely converted to calcium hydroxide. The adsorption experiments showed that the adsorption capacity of AEG900 for fluoride was improved by nearly 29.21% compared with the calcined eggshells without the aging treatment. In the batch experiment, the temperature effect is the most significant for the adsorption process, and nearly a half increment of removal rate is achieved by increasing the temperature by 30 °C. Further research revealed that the adsorption process fitted well with the pseudo-second order model and the Langmuir–Freundlich isotherm model, with a maximum adsorption capacity of 370.15 mg g^−1^. Moreover, precipitation was regarded as the main step for fluoride removal mechanism based on the calculated results of the surface complexation model. X-ray photoelectron spectroscopy (XPS) results showed that the stable fluorite formed *in situ* of AEG900 avoids calcium loss in water. Finally, AEG900 was applied in fluoride removal with real-life groundwater and industrial wastewater, and the results showed that the final fluoride concentration could meet the WHO requirement and industrial wastewater discharge standard.

## Abstract 2

Calcium hydroxide derived from eggshells can remove F^−^ efficiently in water with the adsorption capacity of 370.15 mg g^−1^, and the final concentration can meet the guidelines of the WHO, which is below 1.5 mg L^−1^.

## Graphical abstract

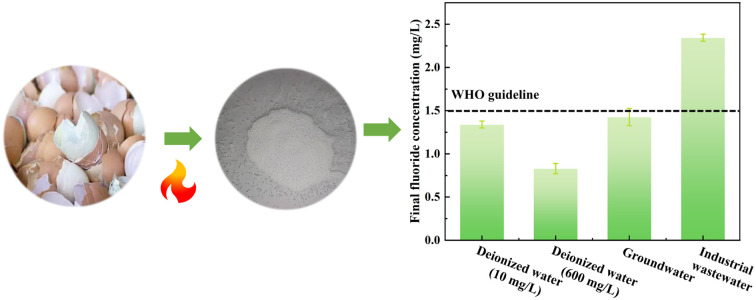

## 1. Introduction

Fluoride generated from natural, anthropogenic, and industrial activities is widespread in different water sources. Especially, flouride will lead to different health effects for humans depending on the concentration. As an essential trace element for bone mineralization and enamel formation, fluoride is beneficial for strengthening the bone and teeth of young children at the level of 0.5 to 1.0 mg L^−1^ in drinking water.^1^ However, the excessive ingestion of fluoride will cause dental fluorosis, skeletal fluorosis, and other diseases; hence, the World Health Organization (WHO) suggests fluoride concentration in drinking water below 1.5 mg L^−1^.^2,3^ Recently, most community water systems in the US have made more serious requirements for fluoride concentration, which has been changed from 0.7 ∼ 1.2 mg L^−1^ to 0.7 mg L^−1^. However, with increasing industrial activities, particularly in the semiconductor manufacturing and smelting industry, thousands of mg L^−1^ of fluoride is discharged into natural water.^4,5^ More than 30 mg L^−1^ in groundwater is detected, which is hazardous for water environment safety. Moreover, according to the statistics from Jadhav *et al.*, China and India are most threatened by fluoride, which nearly occupy 48.30% and 36.62% population of the selected countries.^6^

After realizing the reason for fluorosis, tremendous research studies have been carried out to keep fluoride in water at an acceptable level.^7,8^ The common treatment methods for fluoride removal include chemical precipitation/coagulation, membrane separation, ion exchange, and adsorption. Precipitation/coagulation can be traced back to around 90 years ago. The famous Nalgonda process was developed to remove fluoride in drinking water, which included two steps, the formation of insoluble fluoride with lime and the coagulation treatment with alum. This process is of low cost and has high removal effectiveness, in which the initial concentration of fluoride with 109 mg L^−1^ in groundwater can be reduced to 8 mg L^−1^ after 35 min.^9^ However, in over 10 years of practical applications, some serious drawbacks were found in the Nalgonda process, especially the negative effect of alum and excessive sludge.^10^ Membrane technology is considered a reliable method for fluoride removal in recent years, of which reverse osmosis has become popular to supply safe drinking water and over 95% of fluoride can be removed by membrane separation, and the effluent concentration can be under 0.3 mg L^−1^.^11^ As a relatively new technique, membrane separation involves high costs for skillful operators, film materials, and maintenance, which presents challenges for small-scale water treatment systems. Based on the US Environmental Protection Agency (EPA) report, the removal rate of fluoride can be up to 100% by the adsorption process, which is recognized as one of the best available technologies for fluoride control.^12^

As the EPA recommended adsorbent, activated alumina is widely used to remove fluoride and has been used in large-scale systems.^13,14^ Although this process owns high selectivity and low cost, to achieve a high adsorption performance, a narrow pH range of 5.0–6.0 is required in this process because activated alumina will dissolve at pH < 5, and excessive aluminum will remain in the effluent causing secondary pollution.^15^ At pH > 7, hydroxide and other anions will be more easily adsorbed than fluoride.^16^ On the other hand, while many synthesized adsorbents (mesoporous or modified alumina) display a high removal rate of fluoride, their preparation methods are relatively complex and expensive.^17^ Moreover, few commercial alumina samples can reduce fluoride concentration under the safety limit for drinking water. For example, as Dayananda reported, the commercial alumina only could remove 39% fluoride in water with the initial fluoride concentration of 5 mg L^−1^ after 10 h.^18^ Hence, numerous adsorbents have been developed in recent years to achieve a high-efficient, low-cost, and stable adsorption process for fluoride removal, in which bio-adsorbent is a potential candidate.

Bio-adsorbents are derived from industrial and agricultural wastes, which is a mode of resource utilization for wastes. Eggshells are abundant food waste from daily life, the food industry, and poultry farm. Apart from their use as fertilizers and feed additives, there are still a large number of eggshells without appropriate disposal.^19^ Currently, EPA has ranked eggshells at 15^th^ as one of the environmental problem makers, and more than 100 000 dollars has been paid for the landfill disposal of eggshells in the USA every year.^20^ Due to being rich in calcium carbonate and a natural porous structure, eggshells have a potential application to adsorb heavy metals,^21^ organic dyes,^22^ and anions.^23^ As a calcium-based material, eggshells own high fluoride affinity and biocompatibility. In 2012, Bhaumik *et al.* firstly tried to use eggshells to adsorb fluoride with 5 to 20 mg L^−1^ in water, and the maximum fluoride adsorption capacity was 1.09 mg g^−1^.^24^ They found that the chemical adsorption acted as the main step, and a lower pH would release more Ca^2+^ from calcite on the eggshell surface to benefit fluoride adsorption. While eggshells display a significant advantage for fluoride removal over other bio-adsorbents, there are fewer studies on this system. In 2021, Lee *et al.* restarted this topic, and they found that the CaCO_3_ in natural eggshells changed to CaO by thermal treatment, 70% fluoride could be removed within 15 min, and the maximum adsorption capacity of thermally treated eggshells could reach 258.28 mg g^−1^.^25^ After reviewing the relevant studies about the waste eggshell recycling as bio-adsorbent, most studies were focused on discussing CaO as the main active component to capture anions in water.^26^ In fact, CaO as a solid component in the calcined eggshells is unstable and hygroscopic, leading to an exothermic reaction with water and causing calcium ion loss in water.

Herein, we attempted to recycle Ca(OH)_2_ from calcined eggshells and explore their application as a bio-adsorbent for fluoride removal in water. To our best knowledge, this is the first study reporting Ca(OH)_2_ as the main active component, which is derived from waste eggshells and their application in fluoride removal. The prepared eggshells with different treatment temperatures and aging treatment with moisture were characterized by different physicochemical methods to reveal the effect of composition and structural changes. Then, the batch experiments were conducted to discuss the factors influencing the fluoride removal by eggshells with Ca(OH)_2_ as the main component and found that temperature significantly affected the removal rate rather than pH, as reported in the literature. Moreover, using the equilibrium model calculation and XPS analysis, the fluoride removal mechanism and the contribution of adsorption and precipitation by Ca(OH)_2_ based eggshells were proposed. Finally, the adsorption tests were conducted with real-life industrial wastewater and groundwater to prove their potential for different application backgrounds for fluoride removal.

## 2. Experimental section

### 2.1 Materials

Details of the information for materials are described in ESI.†

### 2.2 Preparation of the adsorbent materials

**Fig. 1:**
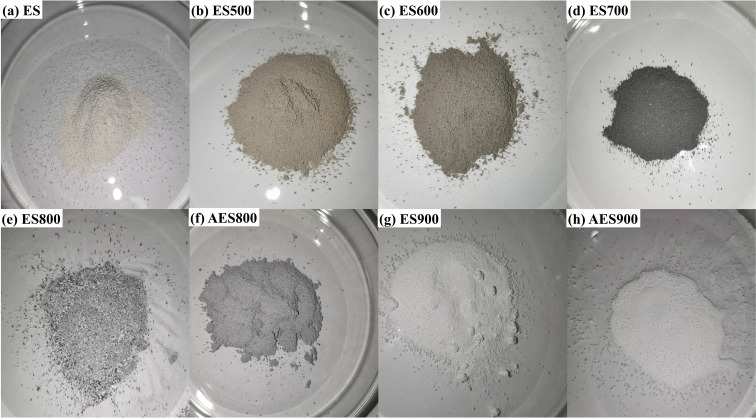
The digital photo of raw eggshells (a), eggshells after heating at 500 (b), 600 (c), 700 (d), 800 (e) and 900 °C (g), and heated eggshells after aging (f) and (h).

The collected eggshells were washed to remove grease and other impurities, and the eggshell membrane was peeled and dried at 100 °C for 24 h. Then, eggshells were ground into small pieces and sieved by a 200-mesh screen to obtain an eggshell powder with a particle size of around 70 μm, as shown in Fig. 1(a). The sieved eggshells were used in the adsorption experiment as the contrast sample named EG. Then, the eggshell powder was heated to 500, 600, 700, 800, and 900 °C in the muffle furnace under air atmosphere for 4 h. As shown in Fig. 1(b)–(f), the obtained samples were denoted as ES500, ES600, ES700, ES800, and ES900, respectively. All calcined samples were stored in a dryer before use in the adsorption experiments, and ES800 and ES900 were selected for further treatment. The aging treatment was conducted in moisture, in which ES800 and ES900 were stored in a box with a relative humidity of 70% for one week. It is worth noting that before the sample characterization and adsorption experiments, the aged samples were dried at 80 °C for 24 h to remove free water.

### 2.3 Adsorption performance

#### 2.3.1 Batch experiment

The adsorption experiments were conducted with fluoride solution in batches to determine the effects of different parameters. A typical batch experiment was carried out as follows. A certain concentration of fluoride solution and adsorbents was added to a 50 mL of polypropylene centrifugal tube. After adjusting the pH, the samples were placed in a shaking incubator operating at 200 rpm and a set temperature for 12 h. After completed adsorption, the samples were centrifuged, and the supernatants were filtered with a 0.45 μm nylon microporous membrane. To measure the residual concentration of fluoride, the filtrate was diluted 50 times to meet the detection limit of ion chromatography (761, Metrohm, Switzerland). The effect of co-existing ions was investigated by adding 10 mM NaHCO_3_, NaSO_4_, K_2_HPO_4_, and NaCl to the initial fluoride solution, respectively. The final concentration of fluoride (*C*_e_ (mg L^−1^)) was recorded, and the removal rate of fluoride and the adsorption capacity of adsorbents were calculated as follows:1
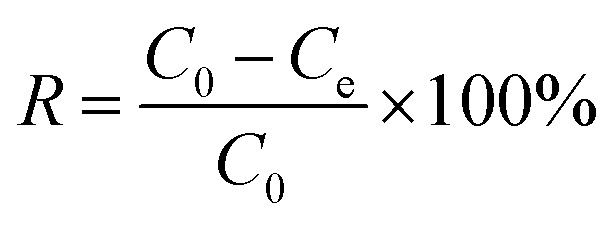
2
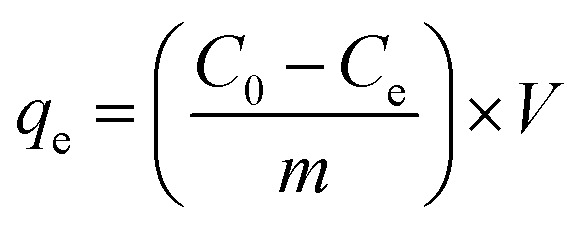
where *C*_0_ (mg L^−1^) is the initial concentration of fluoride, *m* (g) is the mass of adsorbents, and *V* (L) is the solution volume. All experiments were repeated thrice with the error range within 3%, and the average value was discussed.

#### 2.3.2 Adsorption isotherms

To describe the adsorption behavior of adsorbents, a series of adsorption experiments were conducted. Briefly, 1 g L^−1^ adsorbent was added into the fluoride solution with the concentration of 100 to 1000 mg L^−1^ with pH at 7, and then placed in a shaking incubator operating at 200 rpm and 20 °C for 12 h, ensuring to reach the adsorption equilibrium. The residual concentration of fluoride was measured and fitted by the Langmuir (eqn (3)), Freundlich (eqn (4)), and Langmuir–Freundlich (eqn (5)) isotherm models.^27^

Langmuir model:3
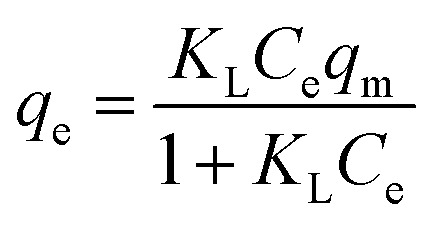


Freundlich model:

4 *q*_e_ = *K*_F_*C*_e_^1/n^

Langmuir–Freundlich model:5
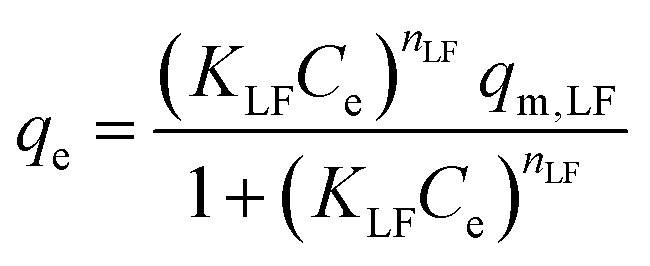
where *q*_m_ (mg g^−1^) and *q*_m,LF_ (mg g^−1^) are the maximum adsorption capacity of the Langmuir model and Langmuir–Freundlich model, respectively, *n* is the Freundlich constant, and *n*_LF_ is the index of heterogeneity varying from 0 and 1. *K*_L_, *K*_F_, and *K*_FL_ are the binding constants for the Langmuir model and Freundlich model, respectively.

#### 2.3.3 Adsorption kinetics

Kinetics experiments were carried out for 1 g L^−1^ adsorbent and 600 mg L^−1^ fluoride solution at pH 7 and 20 °C, and the adsorption time was set from 10 min to 12 h. The residual concentrations of fluoride at different times were measured and fitted by the pseudo-first order (eqn (6)) and pseudo-second order (eqn (7)) equations, respectively.

Pseudo-first-order model:

6 *q*_t_ = *q*_e_[1 − exp(*k*_1_*t*)]

Pseudo-second-order model:7
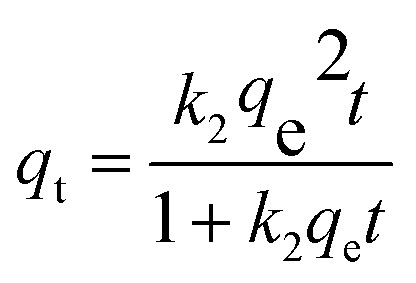
where *q*_t_ (mg g^−1^) is the adsorption amount at different times (*t*), *k*_1_ (h^−1^) is the pseudo-first-order constant, and *k*_2_ (g (mg^−1^ h^−1^)) is the pseudo-second-order constant.

### 2.4 Characterization method

Scanning electron microscopy (SEM, Phenom™ ProX Desktop SEM) was used to observe the surface morphology of eggshells after thermal treatment. The specific surface area and pore size of eggshells were measured by the Accelerated Surface Area and Porosimetry System (SSA-4200, Beijing Builder, China) and calculated based on the Brunauer–Emmett–Teller (BET) and Barrett–Joyner–Halenda (BJH) methods. The Fourier transform infrared spectroscopy (FT-IR, Nicolet is 50, USA) was employed to measure the functional groups on eggshells, and the tablet method with KBr was adopted with the measuring wavelength from 400 to 4000 cm^−1^. The X-ray diffraction (XRD) analysis was conducted to determine the crystal structure and components of the sample by a multi-function diffractometer (DX-2700BH, Haoyuan, China), equipped with a copper (Cu Kα) radiation (*λ* = 1.541 Å) scanning from 10° to 80°. The synchronous thermal analysis (TGA/DSC1, Mettler Toledo, Switzerland) was used to show the mass change of eggshells after thermal treatment at different temperatures. The measurement temperature was from 30 to 1000 °C with an increased rate of 20 °C min^−1^ under air atmosphere. Moreover, X-ray photoelectron spectroscopy (XPS, Thermo Scientific K-Alpha, UK) of eggshells before and after fluoride adsorption was measured.

## 3. Results and discussions

### 3.1 Screening of adsorbent

**Fig. 2:**
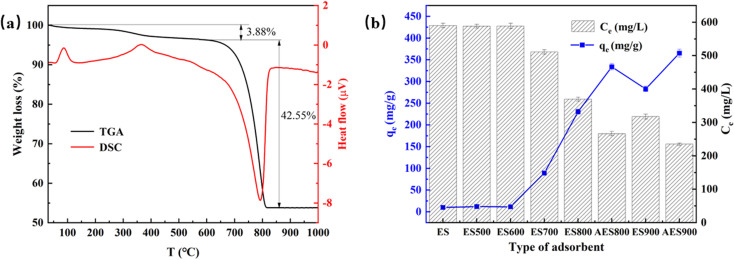
TGA/DSC curves of eggshells (a) and the equilibrium concentration and adsorption capacity of fluoride with different adsorbents (b). ([F^−^]_0_ = 600 mg L^−1^, [adsorbent] = 1 g L^−1^, contact time = 12 h, temperature = 20 °C and pH at neutral).

The effects of calcination temperature and humidity on the preparation of adsorbents are discussed in this section. As shown in Fig. 1, the adsorbents were different visually with different treatment temperatures. With the increase in calcination temperature, the color of eggshells becomes darker and darker until it is black below 700 °C. However, below 800 °C, the color of the powder will turn white, and all of them become white below 900 °C. Aging treatment also can affect the color of the powder, and the white color disappears in AES800. Those color changes reflect the change of components in eggshells after the treatment. Fig. 2(a) shows the TGA curve of eggshells, and a slight mass variation happens before 600 °C with the weight loss of 3.88%, which indicates organic matter in eggshells has become charred, and some of them escaped as CO_2_. Then, a big drop of mass loss is found at about 42.55% until 800 °C, which means the thermal decomposition of CaCO_3_ occurs according to eqn (8). After 800 °C, the eggshell mass remains stable, meaning the decomposition of CaCO_3_ has been completed. Moreover, a distinct inverted peak occurs between 700 to 850 °C in the DSC curve (Left axis in Fig. 2(a)), which is ascribed to the endothermic decomposition of CaCO_3_. It is worth pointing out that the total weight loss of eggshells was 46.43% in this study, which is higher than in a reported work with 40%.^23^ Different calcination environments could cause this difference. In this study, air was chosen because of easier operation, and organic matter would become CO_2_ instead of char.

8 CaCO_3_(s) + heat → CaO(s) + CO_2_(g)

Fig. 2(b) shows the fluoride adsorption on eggshells with different treatment processes. The equilibrium concentration of fluoride decreases from 590.11 to 317.33 mg L^−1^ by increasing the calcination temperature to 900 °C. Obviously, natural eggshells and calcined eggshells obtained below 600 °C are not feasible for fluoride removal, and only 3.68% fluoride is removed for ES600. However, when the calcination temperature increases from 700 to 900 °C, the adsorption capacity of eggshells escalates from 89.07 mg g^−1^ (ES700) to 282.67 mg g^−1^ (ES900), which implies that the component change of eggshells may play the main role for the improvement of fluoride adsorption. A similar tendency is also found in Lee's work.^25^ In their study, the adsorption capacity of ES800 (18.54 mg g^−1^) was similar to ES900 (18.56 mg g^−1^); however, in our study, the adsorption capacity of ES900 is obviously larger than that of ES800 (230.42 mg g^−1^). Apart from the effect of different calcination conditions, BET results could explain this discrepancy to a certain extent. Indeed, the specific surface area (SSA) of ES900 (2.785 m^2^ g^−1^) was more than twice that of ES800 (1.08 m^2^ g^−1^), which could provide more active sites for fluoride. Moreover, aged eggshells display higher adsorption capacity for fluoride, which increases to 333.70 mg g^−1^ for AES800 and 365.24 mg g^−1^ for AES900, respectively. This enhancement is considerable, in which the equilibrium concentration drops to 234.76 mg L^−1^ for AES900, meaning 61% fluoride could be removed even with a high initial concentration. Eqn (9) is the main reaction that happens in the aging stage in eggshells, where CaO adsorbing moisture from air would convert to hydrated lime, which is more favorable for fluoride removal.

9 CaO(s) + H_2_O(g) → Ca(OH)_2_(s)

**Fig. 3:**
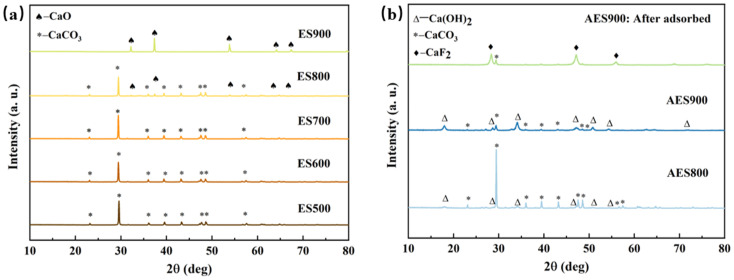
XRD patterns of heated eggshells with different temperatures (a) and aged eggshells before and after adsorption of fluoride (b).

In order to identify the components formed in the eggshells at different calcination temperatures, XRD patterns of each adsorbent were obtained, as shown in Fig. 3(a). The results confirm that when the calcination temperature is below 700 °C, the major component in the samples is calcite (JCPDS No. 05-0586). When eggshells are heated under 800 °C, lime is formed in ES800 (JCPDS No. 37-1497). By comparing the XRD patterns of ES800 and ES900, it can be deduced that almost all CaCO_3_ converts to CaO at 900 °C because only the pattern of lime can be found in the ES900 XRD pattern, and the same result was reported in Risso's study, who attempted to obtain CaO catalysts from eggshells at 900 °C.^28^ Unlike that described in Lee's study, Ca(OH)_2_ is not present in fresh ES800 and ES900 due to strict dry storage conditions.^25^ Conversely, Ca(OH)_2_ (JCPDS No. 04-0733) is formed after storing in humid air due to eqn (9), and CaO almost disappears in aged samples, as shown in Fig. 3(b). Moreover, the proportion of Ca(OH)_2_ in AES900 is obviously higher than that in AES800, which could be the reason for a higher adsorption capacity for fluoride for AES900, as mentioned in Fig. 2(b). Panagiotou *et al.* suggested that Ca(OH)_2_ was more soluble than CaO or CaCO_3_, so soluble Ca in water would be more reactive with the target ions, and the reason for the removal of ions was precipitation rather than adsorption.^29^ To prove that the adsorption happened on eggshells, the XRD result of AES900 after fluoride adsorption to equilibrium is shown in Fig. 3(b), and CaF_2_ (JCPDS No. 35-0816) can be found from the main diffraction patterns, which indicates that the ion exchange happens and generates fluorite on the surface of AES900 based on eqn (10).^30^ Moreover, the peak of Ca(OH)_2_ almost disappears, but the peak of CaCO_3_ can still be found in the XRD spectrum of AES900 after adsorption. Hence, Ca(OH)_2_ plays the main role in fluoride removal, and aging treatment with humid air is very important to improve the adsorption capacity of calcined eggshells.

10 Ca(OH)_2_(s) + 2F^−^ → CaF_2_(s) + 2OH^−^

**Fig. 4:**
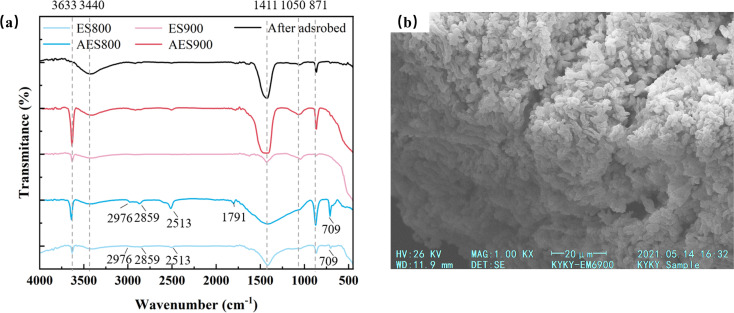
FT-IR spectra for different adsorbents and AEG900 after adsorbed fluoride (a) and SEM morphological images for AEG900 (b).

To obtain more information about the structure of adsorbents, the FT-IR spectra of adsorbents thermally treated above 800 °C were measured, as shown in Fig. 4(a). Before adsorption, the exhibited adsorption peaks include 3633, 3440, 1411, 1050, and 871 cm^−1^ for all samples. Among them, the sharp peak at 3633 cm^−1^ and the broad peak at 3440 cm^−1^ are attributed to the bending vibrations of –OH from Ca(OH)_2_ and crystalline hydrate, respectively.^31,32^ A higher relative intensity peak at 3633 cm^−1^ can be found in the aged samples but not in calcined samples at 800 or 900 °C, which is in agreement with the XRD result that more Ca(OH)_2_ is generated by aging treatment. The peaks at 1411 and 1050 cm^−1^ and the peaks at 709 and 2513 cm^−1^ in ES800 and AES800 belong to the vibrations of C–O bonds.^33^ Moreover, the peaks at 2976, 2859, and 1791 cm^−1^ are attributed to the C=O bonds from CO_3_^2−^, and the above-mentioned bands indicate the presence of CaCO_3_ in ES800 and AES800.^31^ However, the peaks belonging to CaCO_3_ are not found in the sample calcined 900 °C, which could be explained by a lower relative peak intensity. Moreover, the sharp bands at 871 cm^−1^ correspond to the Ca–O bond, and the broad peaks at 498 cm^−1^ are from the Ca=O bonds.^34^ After adsorption, the peak of –OH bond at 3633 cm^−1^ disappears in AES900, which indicates that the active site of Ca–OH on the surface reacted with F^−^. The peak of Ca–O bond at 871 cm^−1^ becomes weak and the peak of Ca=O bonds disappears and these indicate the reaction happened between Ca and F. The change of AES900 before and after adsorption confirms the key role of Ca(OH)_2_ for fluoride adsorption again.^35^ Furthermore, the SEM image of AES900 was taken, as shown in Fig. 4(b). After calcination, the pore size of the eggshell powder became smaller (<5 μm), which is also reflected in the BET data (SSA increased from 0.515 to 2.785 m^2^ g^−1^). Moreover, the common pores from natural eggshells could not be found in the SEM image because the average pore size was 12.8 nm for AES900.^36^

Moreover, the final pH and stability of adsorbents also were discussed, and the results are listed in Table S1.† During the adsorption experiment, the initial pH was around 6.66, and the final pH was around 10.51 for natural eggshells and eggshells with calcination temperature below 700 °C. There is no obvious difference in the final pH for eggshells with or without aging treatment, which are all around 12.01. The final pH increase is a common phenomenon in Ca-based materials for fluoride removal and also happens in the case of sepiolite.^37^ The stability of adsorbents was evaluated by measuring the weight loss of adsorbents during the adsorption experiment and calculated as TDS (%) shown in Table S1.† TDS displayed the proportion of weight loss of adsorbents to raw adsorbents after adsorption of fluoride. From the data in Table S1,† it is clear that the stability of natural eggshells and calcined eggshells obtained below 700 °C are very weak, and more than 50% weight loss happened in the fluoride adsorption process. Combined with pH change, it could be deduced that CaCO_3_ as the main component in these materials would dissolve in water with a weak acidic condition (pH = 6.66) causing mass loss, and the increase in the final pH reflects the consumption of H^+^ during the dissolution of calcite.^38,39^ Moreover, eggshells obtained by thermal treatment above 800 °C show higher stability. The values of TDS for EG800 and EG900 are 14.33% and 10%, respectively. As the main reason, CaO in those materials reacts with water to form Ca(OH)_2_, and meanwhile, Ca dissolves in water, causing mass loss, and Santos *et al.* gave a similar explanation for the weight loss of EG800.^23^ In contrast, the stability of aged eggshells is the best, and the values of TDS are 4.67% and 3.00% for AEG800 and AEG900, respectively. In this system, the reaction between fluoride and Ca(OH)_2_ might have a major role, and forming CaF_2_ covered on the surface of eggshells would prevent Ca loss.

Hence, above all, AES900 displays an excellent adsorption capacity and better stability for the removal of high concentrations of fluoride in water, and the adsorption performance of AEG900 with different effects will be discussed in the next section comprehensively.

### 3.2 Batch adsorption experiments

#### 3.2.1 Effects of pH, temperature, other ions on adsorption

**Fig. 5:**
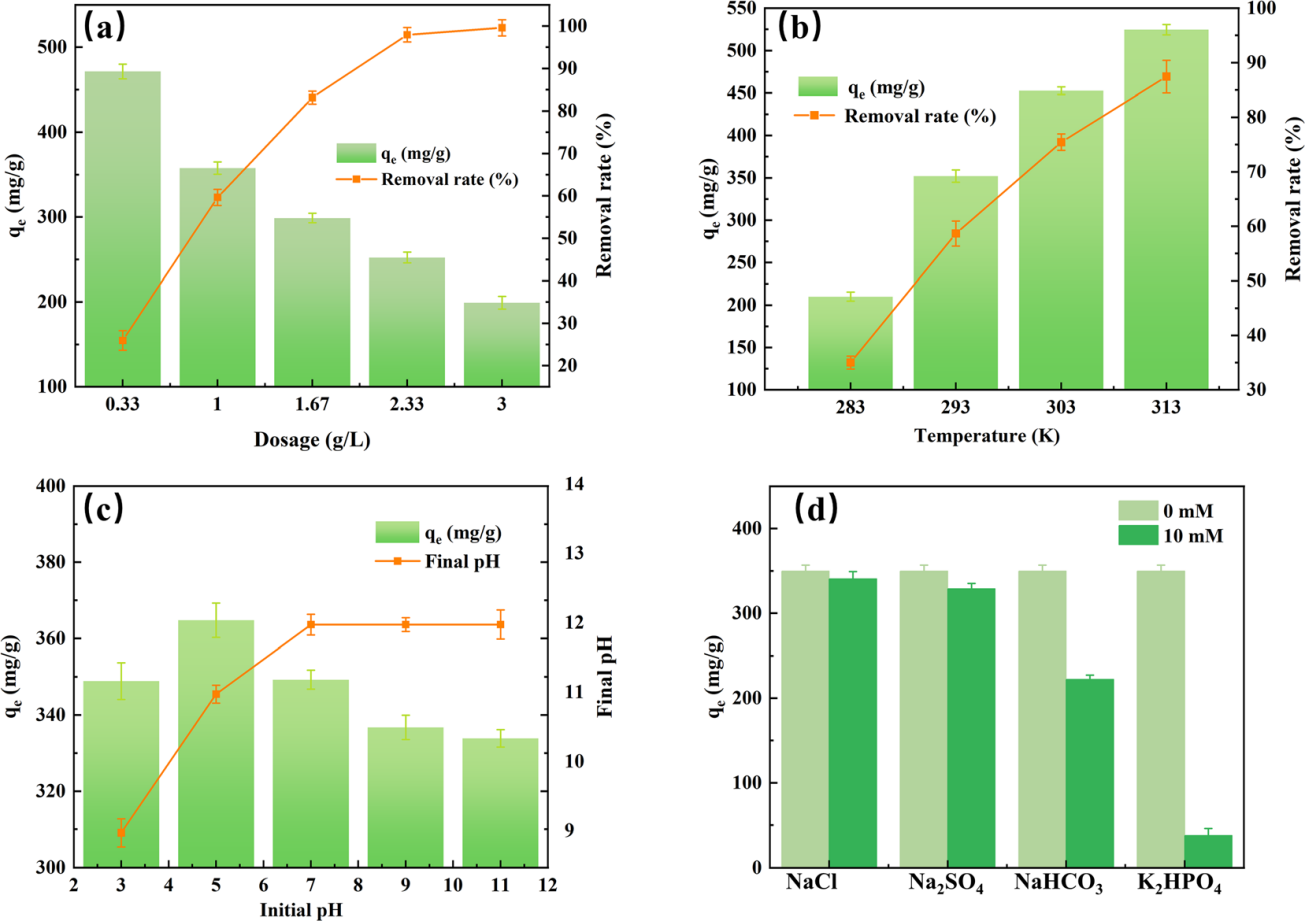
Effects of dosage (a), temperature (b), pH (c), and other ions (d) for the adsorption performance of fluoride onto AES900. ([F^−^]_0_ = 600 mg L^−1^, contact time = 12 h, [adsorbent] = 0.33–3 g L^−1^, temperature = 10–40 °C, initial pH = 3–11).

The adsorbent dosage, pH, temperature, and other ions as the main operating parameters were discussed, and the results are shown in Fig. 5. Fig. 5(a) shows that with an increase of AES900 dosage from 0.33 to 3 g L^−1^, the removal rate of fluoride increases from 25.92% to 99.86% for 600 mg L^−1^ fluoride solution. It is worth noting that the increment rate gradually decreases, especially between the dosage of 2.33 (97.93%) and 3 g L^−1^ (99.86%), in which the removal rate of fluoride only increases by 1.93%. It can be explained by the weak driving force for adsorption in a low concentration of fluoride (the final concentration is 10.55 and 0.83 mg L^−1^, respectively).^40^ On the other hand, the adsorption capacity decreases with increasing dosage of AES900, which means the increase in unsaturated active sites, and excess active sites will not improve fluoride adsorption significantly.^41^ Moreover, compared with other studies, the adsorption capacity is 199.72 mg g^−1^ for 3 g L^−1^ of AES900, which is higher than other adsorption systems for fluoride removal. For example, the adsorption capacity of CaO_20_@Al_2_O_3_ with 3 g L^−1^ for 600 mg L^−1^ fluoride was about 120 mg g^−1^.^42^

Fig. 5(b) shows the effect of temperature on fluoride adsorption. With increasing temperature from 10 to 40 °C, the removal rate of fluoride increases from 35.0 to 87.44%. This increment is significant (nearly 52.44%) within a 30 °C increase compared with other studies.^43,44^ This is probably because the increasing temperature could increase the diffusion of fluoride to the adsorbent and enhance the reaction between Ca(OH)_2_ and F^−^.

Fig. 5(c) shows the effect of initial pH for fluoride adsorption and final pH in solution. With an increase in pH, the adsorption capacity increases from 348.89 mg g^−1^ at pH 3, peaking at 364.77 mg g^−1^ at pH 5 and then decreases to 333.86 mg g^−1^ at pH 11. This tendency is similar to Saini's work and their plots of the speciation curve of fluoride in solution at different pH could be helpful in understanding this phenomenon.^43^ Firstly, at pH below 3, more than half of fluoride will present as HF, which will further form a stable association to reduce the concentration of free fluoride ions.^45^ When pH increases to 5, most of the fluoride will present as free fluoride ions, which is favorable for reaction with the adsorbent. On the other hand, in weak acidic conditions, more active sites will explore and carry a positive charge, which will capture fluoride ions easily by electrostatic attraction.^39^ At a basic pH, while all the fluorides are present as free ions, the adsorption capacity still decreases, which is mainly due to the physical changes on the surface of AES900. In alkaline conditions, the surface of the adsorbent will carry a negative charge, which will repel the fluoride ions.^39^ Moreover, OH^−^ can act as a competitor for fluoride adsorption, and more OH^−^ will occupy the active sites, leading to a decrease in adsorption capacity for fluoride.^46^

Apart from fluoride ions, other ions also exist in the natural water and also can adsorb on eggshells acting as a competitor in the fluoride adsorption process. Herein, chloride, bicarbonate, sulfate, and hydrogen phosphate as typical competitive ions were discussed. Fig. 5(d) shows the effects of other ions on the adsorption of fluoride. It can be found that co-existing ions have negative effects on fluoride adsorption and the adsorption capacity of fluoride decreases in the order of HPO_4_^2−^ > HCO_3_^−^ > SO_4_^2−^ > Cl^−^, which is similar to other studies.^25,47^ Among them, the negative effects of chloride and sulfate are not significant, and the adsorption capacities of fluoride only decrease by 2.42% and 5.77%, respectively. Bicarbonate and hydrogen phosphate have remarkable inhibitive effects on fluoride adsorption, and the adsorption capacities of fluoride decrease by about 36.32% and 89.06%, respectively. The main reason for the competitive adsorption of bicarbonate is the increase in pH, which is adjustable before the adsorption process. However, the negative effect of hydrogen phosphate is inescapable, and to ensure the feasibility of fluoride removal, dephosphorization treatment for the inflow is necessary.

#### 3.2.2 Adsorption kinetics

**Fig. 6:**
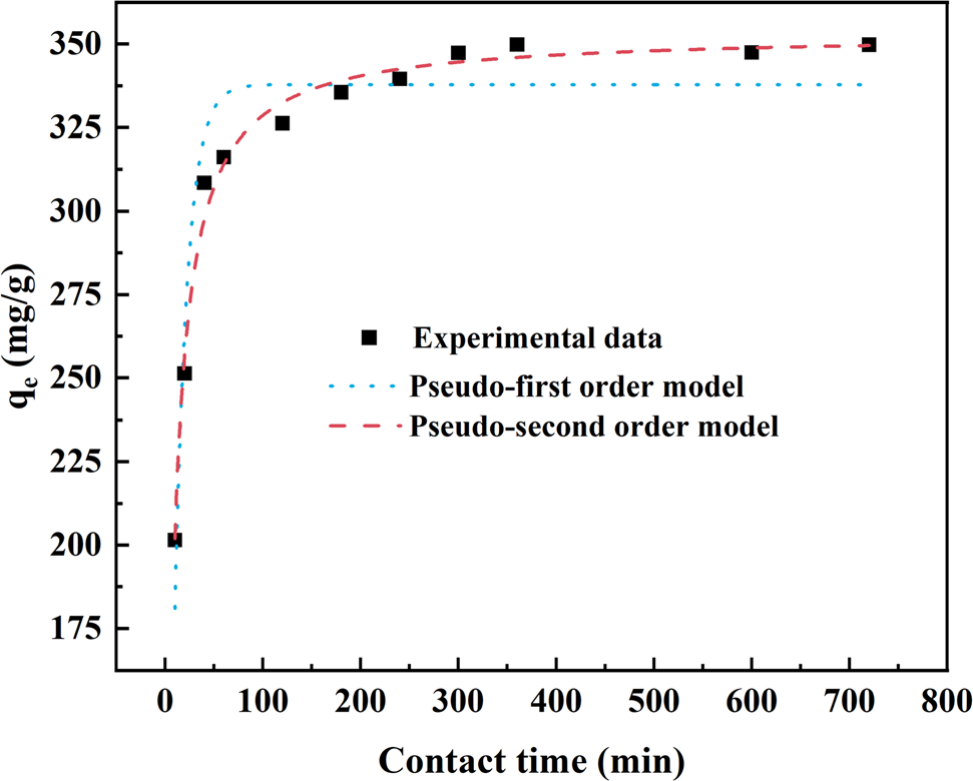
Kinetic model fitting results for fluoride adsorption by AES900. ([F^−^]_0_ = 600 mg L^−1^, [adsorbent] = 1 g L^−1^, temperature = 20 °C and pH at neutral).

In order to understand the rate controlling step of mass transfer and the effect of contact time for fluoride adsorption on AEG900, the pseudo-first and pseudo-second order models were applied to determine the adsorption rate of fluoride on AEG900. Fig. 6 depicts the measured adsorption capacity as a function of contact time and their fitting curves with two kinetic models. The result shows that the adsorption capacity increases sharply within the first 100 min, and active sites are fast occupied by fluoride. Then, with the decrease of active sites, the adsorption capacity increases slowly near the adsorption equilibrium until 720 min. Based on the *R*^2^ listed in Table 1, pseudo-second order model is better for describing the adsorption process of fluoride on AES900 (*R*^2^ > 0.989), and the calculated *q*_e_ is similar to the experimentally measured value with a relative error of 3%. The pseudo-second order model assumes that chemisorption is the controlling step, which describes the sharing or exchanging charge between adsorbent and adsorbate to generate new compounds. Hence, precipitation, diffusion, and surface adsorption could happen in the adsorption of fluoride onto AEG900. Moreover, the experimental data also is fitted in the intra-particle diffusion model (eqn (S1)†), and the fitting result is shown in Fig. S1.† It can be found that the adsorption process can be divided into three continuous steps involving surface diffusion, intra-particle diffusion, and the equilibrium stage of adsorption and desorption. None of these three plots passes through the original point (*C*_1_ ≠ 0), meaning the internal diffusion is not a single step controlling the whole adsorption process. It also proves that the adsorption of fluoride onto AES900 involves more than one mechanism.^46^

**Table 1:** Kinetic model parameters for fluoride adsorption onto AES900

Model	Pseudo-first order model			Pseudo-second order model		
	*k*_1_ (min^−1^)	*q*_e_ (mg g^−1^)	*R* ^2^	*k*_2_ (g mg^−1^ min^−1^)	*q*_e_ (mg g^−1^)	*R* ^2^
Parameters	0.0767	337.834	0.923	0.000378	353.171	0.989

#### 3.2.3 Adsorption isotherm

**Fig. 7:**
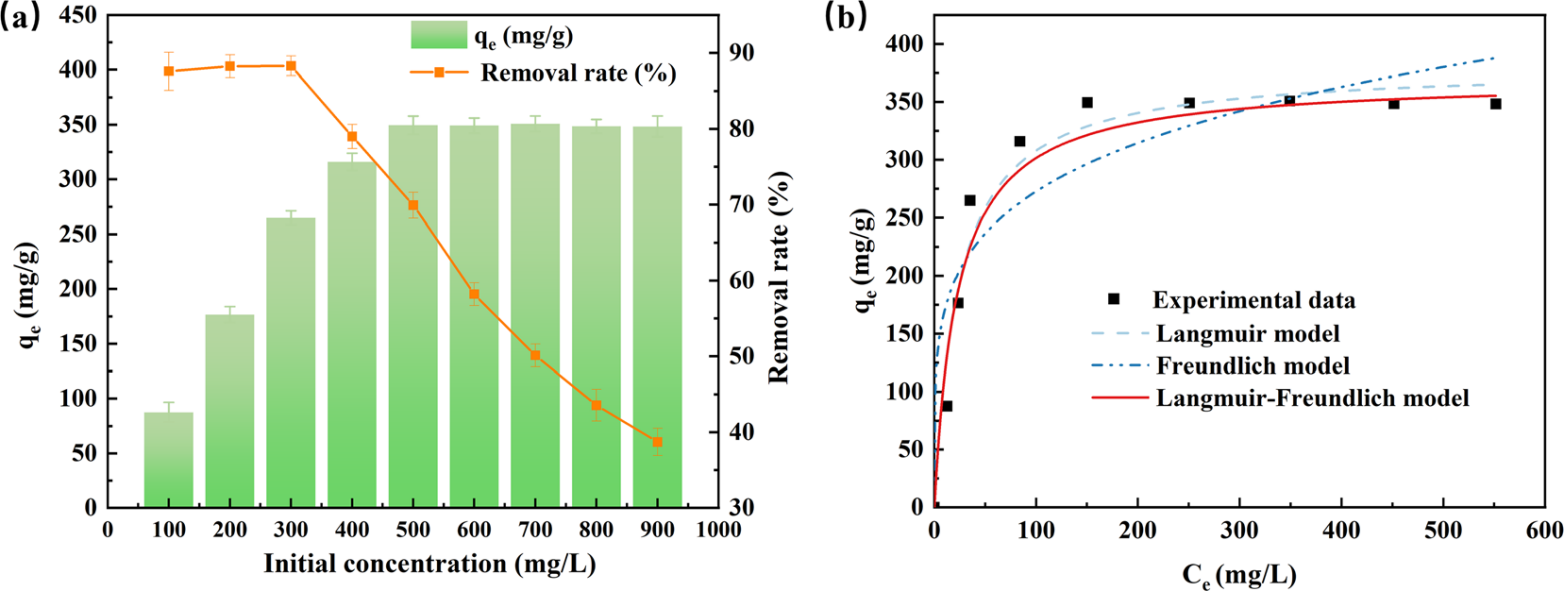
Fluoride adsorption as a function of initial concentration by AEG900 (a) and the isotherm models fitting results (b). ([F^−^]_0_ = 100–900 mg L^−1^, [adsorbent] = 1 g L^−1^, contact time = 12 h, temperature = 20 °C and neutral pH).

The effect of initial concentration for fluoride adsorption on AEG900 was investigated, as shown in Fig. 7(a). The adsorption capacity increases firstly from 87.57 to 349.56 mg g^−1^ as the initial concentration increases from 100 to 500 mg L^−1^, and then with further increasing initial concentration, the adsorption capacity remains constant. Inversely, the removal rate decreases from 88.28% to 38.71%, with the initial concentration increasing from 300 to 900 mg L^−1^. A slight increase in removal rate occurs within the initial concentration between 100 to 300 mg L^−1^, which means a low initial concentration may affect the fluoride adsorption due to the lack of driving force, so for the removal of fluoride with a low initial concentration in water, the feasibility of AEG900 should be discussed further.

Moreover, the experimental data is fitted in isotherm models, including Langmuir, Freundlich, and Langmuir–Freundlich models, as shown in Fig. 7(b), and the obtained parameters are listed in Table 2. Clearly, the Freundlich model is not suitable for describing the fluoride adsorption onto AES900 with *R*^2^ > 0.87. While the *R*^2^ of fitting the Langmuir curve is higher than 0.94, the simulated *q*_m_ is 380.93 mg g^−1^ with a 4.11% relative error for the experimental value. The fitting result by the Langmuir–Freundlich model has the highest correlation (*R*^2^ > 0.96), and the calculated *q*_m,LF_ only has a 1.37% relative error for experimental value. Langmuir–Freundlich model is favorable for heterogeneous systems with the variable density function, which is determined by *n*_LF_.^48^ If *n*_LF_ equals 1, the adsorbent material is homogeneous, and the Langmuir–Freundlich model is reduced to the Langmuir model. In this study, *n*_LF_ is 0.34, indicating AEG900 is a heterogeneous material to adsorb fluoride.

**Table 2:** Isotherm model parameters for fluoride adsorption onto AES900

Model	Langmuir			Freundlich			Langmuir–Freundlich			
	*q*_m_ (mg g^−1^)	*K*_L_ (L mg^−1^)	*R* ^2^	*K*_F_ (mg g^−1^)	*n* (g L^−1^)	*R* ^2^	*q*_m,LF_ (mg g^−1^)	*K*_LF_ (L mg^−1^)	*n*_LF_	*R* ^2^
Parameters	380.93	0.04	0.94	105.46	4.85	0.87	370.15	0.04	0.34	0.96

### 3.3 Removal mechanism analysis

#### 3.3.1 Surface complexation modeling

**Fig. 8:**
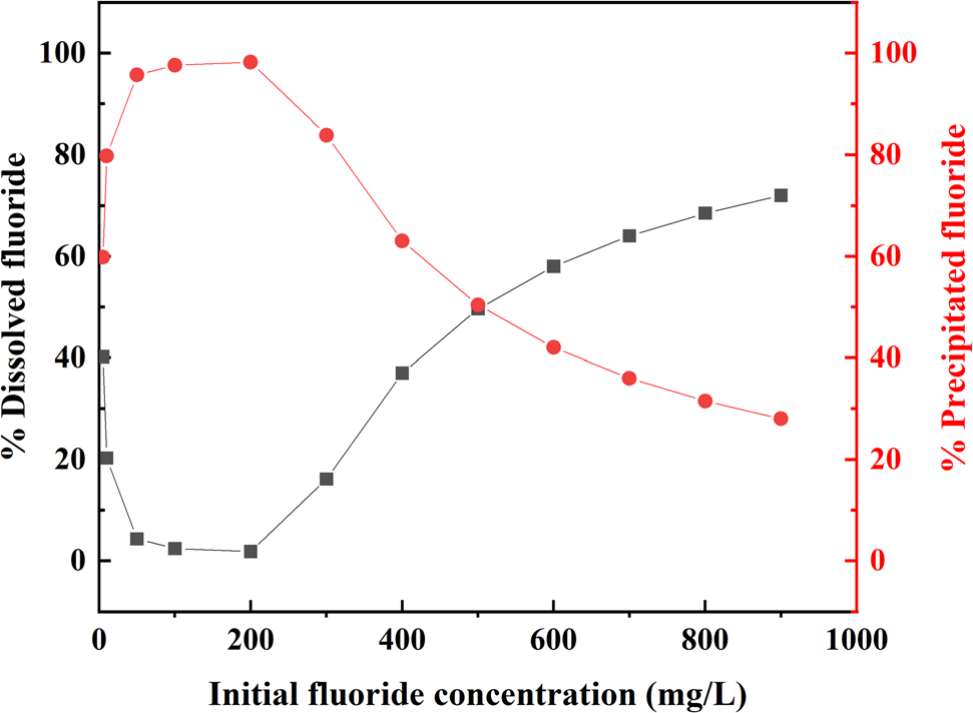
Equilibrium mass distribution of fluoride between the dissolved and precipitated phase predicted by Visual MINTEQ 3.1.

According to the discussion result of batch adsorption experiments, the kinetic analysis points that the adsorption process of fluoride on AEG900 fitted well with the pseudo-second order models, which means adsorption and co-precipitation both participate in the removal of fluoride. Moreover, the adsorption isotherm curve fits well with the Langmuir–Freundlich model, indicating the adsorption process is monolayer adsorption, and the adsorbent is a heterogeneous material. Therefore, the contribution degree of adsorption and precipitation should be discussed first to reveal the whole removal mechanism of fluoride by AEG900. Herein, the Visual MINTEQ 3.1 software was employed to calculate the chemical equilibrium to predict the co-precipitation behavior between fluoride and calcium ions.^46^ Firstly, the set concentration of fluoride ions and the experimentally obtained total dissolved concentration of calcium ions were used as inputs for an aqueous system. The total dissolved concentration of calcium ions was measured as 266 mg L^−1^ for 1 g L^−1^ AEG900. Then, a solid phase in this aqueous system by the three planes model (Calcite-CDM) to form a complexation surface was added. The input parameters for the complexation surface are listed in Table S2,† including the SSA, dosage, and equilibrium pH from the experimental data. As one of the output results, saturation indices (SI) for minerals are obtained to predict the formation potential of possible components in this system, of which the positive value means oversaturation, zero means apparent equilibrium, and the negative value means undersaturation. When the initial fluoride concentration is 600 mg L^−1^, the SI of fluorite, lime, and portlandite is 0, -16.19, and −6.00, respectively, which indicates that only fluorite is a possible co-precipitated product. Moreover, the equilibrium mass distribution of fluoride and calcium ions between dissolved and precipitated phases predicted by Visual MINTEQ 3.1 are summarized in Table S3,† of which the equilibrium mass distribution of fluoride is plotted in Fig. 8. As shown in Fig. 8, when the initial fluoride concentration is between 50–200 mg L^−1^, more than 95.67% fluoride will be precipitated with calcium to form fluorite. As fluoride concentration increases, the proportion of dissolved fluoride will increase from 1.83% to 72.0% due to the excess fluoride ions, and the proportion of precipitated fluoride will decrease correspondingly. By combining the predicted and experimental data, the amount of fluoride at different phases is summarized in Table 3. Obviously, the dominant mechanism of fluoride removal for AEG900 is co-precipitation when the initial fluoride concentration is below 200 mg L^−1^. Fluoride ions will form CaF_2_ preferentially, with sufficient calcium ions dissolving from AEG900. Moreover, the predicted precipitation value is higher than the experimental value because the adsorption equilibrium is hard to reach at a low initial concentration within 12 h. When the initial fluoride concentration increases over 200 mg L^−1^, the co-precipitation and adsorption will both act as the removal mechanism for fluoride, in which the co-precipitation is predominant. We also measured the calcium concentration in solution for the initial fluoride concentration at 600 mg L^−1^ and found that the residual calcium concentration was less than 1.33 mg L^−1^. Hence, it proves again that most of the calcium ions will react with fluoride to form co-precipitated products.

**Table 3:** Fluoride distribution between the dissolved, precipitated, and adsorbed phases based on the predicted and experimental results

Initial fluoride concentration (mg L^−1^)	Experimental result	Visual MINTEQ prediction		Precipitated fluoride^a^ (mg L^−1^)	Adsorbed fluoride^b^ (mg L^−1^)
	Final fluoride concentration (mg L^−1^)	Total dissolved fluoride (mg L^−1^)	Total precipitated fluoride (mg L^−1^)		
5	2.12	2.01	2.99	2.88	0.00
10	4.77	2.02	7.98	5.23	0.00
50	7.53	2.16	47.85	42.47	0.00
100	12.42	2.40	97.61	87.58	0.00
200	23.50	3.66	196.35	176.50	0.00
300	35.15	48.33	251.69	251.67	13.18
400	84.03	148.07	251.96	251.93	64.04
500	150.43	248.06	251.98	251.94	97.63
600	250.75	348.06	252.00	251.94	97.31
700	349.13	448.06	252.00	251.94	98.93
800	451.47	548.06	252.00	251.95	96.58
900	551.62	648.07	252.00	251.93	96.45

a Calculated from initial fluoride concentration minus final fluoride concentration and the calculated adsorbed fluoride concentration.

b Calculated from predicted total dissolved fluoride minus final fluoride concentration in the experiment. If the result is negative, the value of adsorbed fluoride is regarded as 0.

#### 3.3.2 XPS analysis

**Fig. 9:**
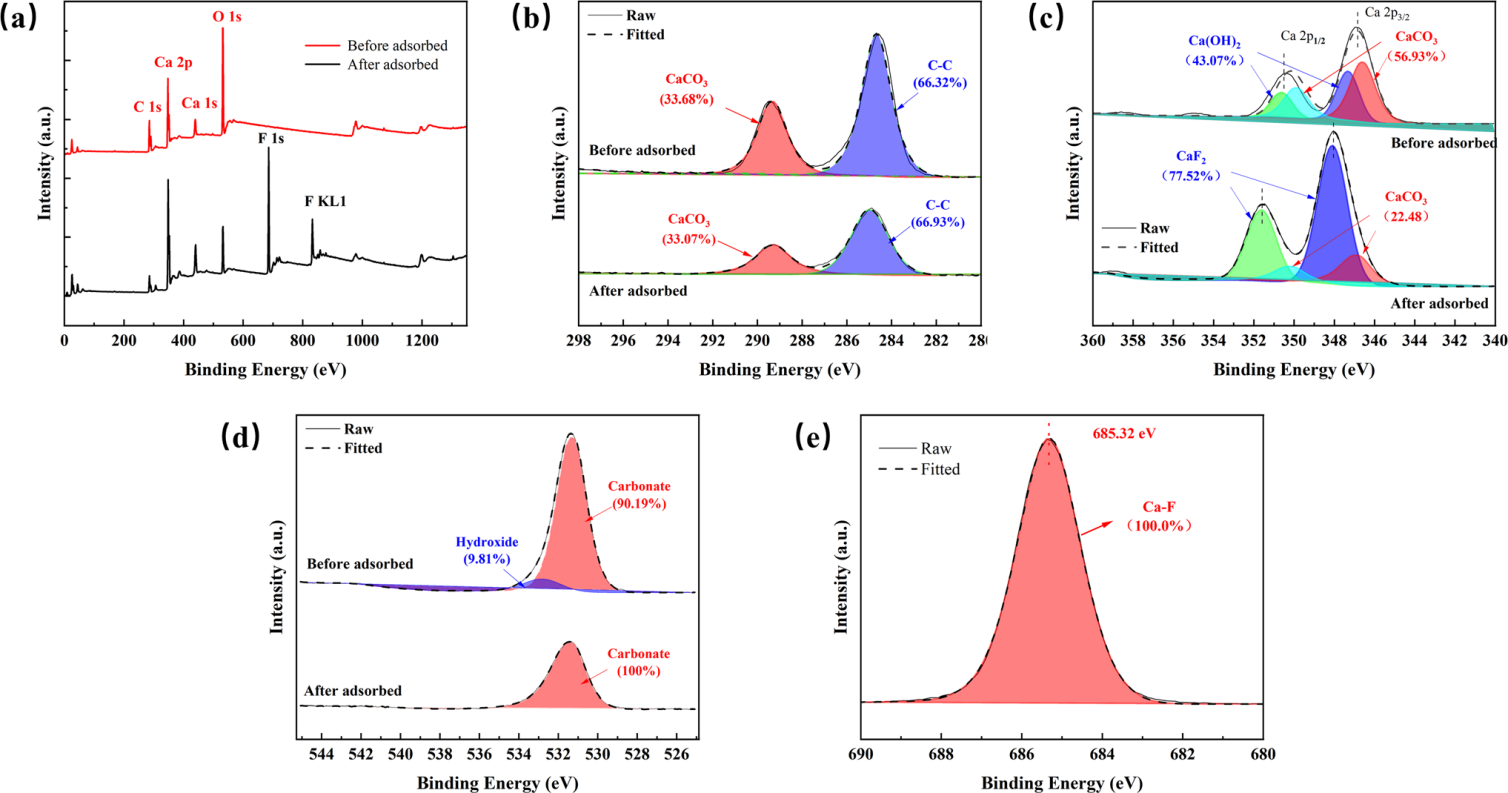
XPS curves of AEG900 before and after fluoride adsorption (a), C 1s spectrum (b), Ca 2p spectrum (c), O 1s spectrum (d) for AEG900 before and after fluoride adsorption and F 1s spectrum (e) for AEG900 after fluoride adsorption.

Based on the analysis result of surface complexation modeling, precipitation plays a predominant role for fluoride removal. As previously reported, while quick lime could remove fluoride efficiently under suitable alkaline conditions, the separation and disposal of produced sludge will cause some extra load for the treatment system.^49^ However, based on the XRD results shown in Fig. 2(a), fluoridate precipitation could happen on the eggshell surface. To further reveal the removal mechanism of fluoride by eggshells, XPS analysis was carried out for AEG900 before and after the adsorption of fluoride. As shown in Fig. 9(a), besides Ca 2p, O 1s and C 1s signals can be found in the low energy regions of the full XPS spectra in the two samples, and F 1s signal can be clearly found in AEG900 after fluoride removal, which confirms the migration of fluoride from water to AEG900. The intensity of the F 1s signal also is considerable compared with that of other elements, and by a rough calculation, the proportion of fluoride atoms is about 35.36% in AEG900. Moreover, the F 1s spectra were fitted and the binding energy of the F 1s signal is 685.32 eV, as shown in Fig. 9(e), while the binding energy of NaF (684.50 eV) is lower, which implies the reaction between calcium and fluoride ions to form CaF_2_ rather than the crystallization of NaF onto AEG900.^32^ The Ca 2p spectra also was fitted and decomposed into different species as shown in Fig. 9(c). It is clearly seen that the peaks of Ca 2p_1/2_ and Ca 2p_3/2_ shift before and after the fluoride removal due to the higher electronegativity of fluoride. For raw AEG900, the binding energies of Ca 2p in 347.32 and 350.62 eV belongs to Ca(OH)_2_ and those of 346.58 and 349.88 eV belong to CaCO_3_.^50^ Based on the fitted result, the contribution of Ca(OH)_2_ is 43.07% and that of CaCO_3_ is 56.93%, which is in accordance with the XRD result as shown in Fig. 2(b). After fluoride removal, the Ca 2p signal can be divided into two species, including CaCO_3_ with the binding energies of 346.85 and 350.15 eV and CaF_2_ with the binding energies of 348.09 and 351.63 eV.^25^ It can be inferred that Ca(OH)_2_ plays the main role in removing fluoride by precipitation, while CaCO_3_ also is involved in the reaction. To be specific, the contributions of CaF_2_ and CaCO_3_ were 77.52% and 22.48%, respectively.

Moreover, as shown in Fig. 9(b), the change of C 1s spectra for AEG900 before and after adsorption are slight, and the two peaks belong to CaCO_3_ and C–C at 289.26 and 284.98 eV, respectively. For the O 1s spectra in Fig. 9(d), the peaks of metal carbonate and metal hydroxide can be decomposed with the binding energies at 531.34 and 532.88 eV, respectively, for raw AEG900. However, after the removal of fluoride, the metal hydroxide peak disappeared in the O 1s spectra, implying the dissociation of hydroxide accompanied by calcium dissolution from AEG900 in water. This conclusion is consistent with the FT-IR result of AEG900, as shown in Fig. 3, in which a sharp peak of –OH groups at 3633 cm^−1^ disappeared after the removal of fluoride. In conclusion, XPS analysis indicates that a strong interaction occurs between calcium and fluoride to form a stable component on the adsorbent, in which Ca(OH)_2_ is consumed preferentially compared with CaCO_3_.

#### 3.3.3 Mechanism of fluoride removal by AEG900

**Scheme 1:**
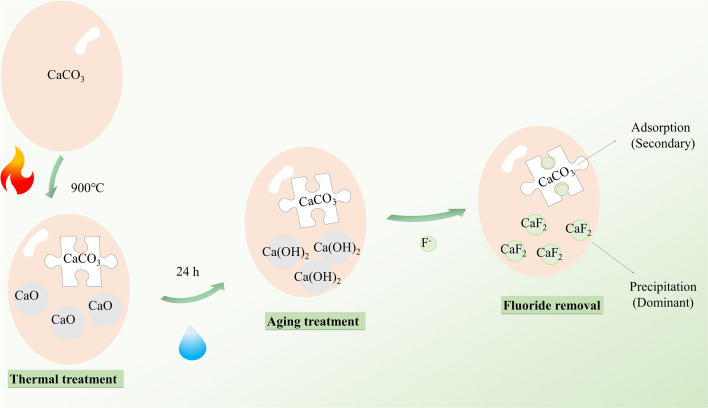
The removal mechanism of fluoride by AEG900.

On the basis of material characterization, the batch adsorption experiment, and model analysis, the removal mechanism of fluoride by AEG900 is shown in Scheme 1. By thermal treatment, part of CaCO_3_ in eggshells will convert to CaO, which could improve the dissolution of Ca ions to capture fluoride ions in water. However, compared with Ca(OH)_2_, the ability to release Ca ions for CaO is weaker, so by the aging treatment under a humid environment, CaO can convert to Ca(OH)_2_ with higher dissolution capacity. Hence, unlike the common adsorbent recovery from eggshells, Ca(OH)_2_ becomes the core component in AEG900. Combined with the results in the single-factor experiment, this adsorption process fits with the pseudo-second kinetic and Langmuir–Freundlich models well, indicating the presence of chemisorption and that the adsorbent material is heterogeneous. By the mass equilibrium analysis, dissolution, co-precipitation, and adsorption are involved in the removal process of fluoride by AEG900. Based on the surface complexation modeling predicted, precipitation is the dominant mechanism for fluoride removal. On the other hand, while the difference in SSA for eggshells treated under different conditions is slight, the removal efficiency of fluoride is enhanced by the aging treatment, indicating that adsorption is not a key point for fluoride removal. So, based on model prediction, adsorption only works under a high fluoride concentration. Moreover, the *in situ* precipitation of fluorite is found based on the characterization results of XPS and XRD. Correspondingly, the mass loss of AEG900 before and after use is negligible. Hence, the removal mechanism of fluoride by AEG900 is precipitation and adsorption, in which *in situ* precipitation preferentially occurs on the surface of AEG900.

### 3.4 Evaluation of fluoride removal in real-life water

**Fig. 10:**
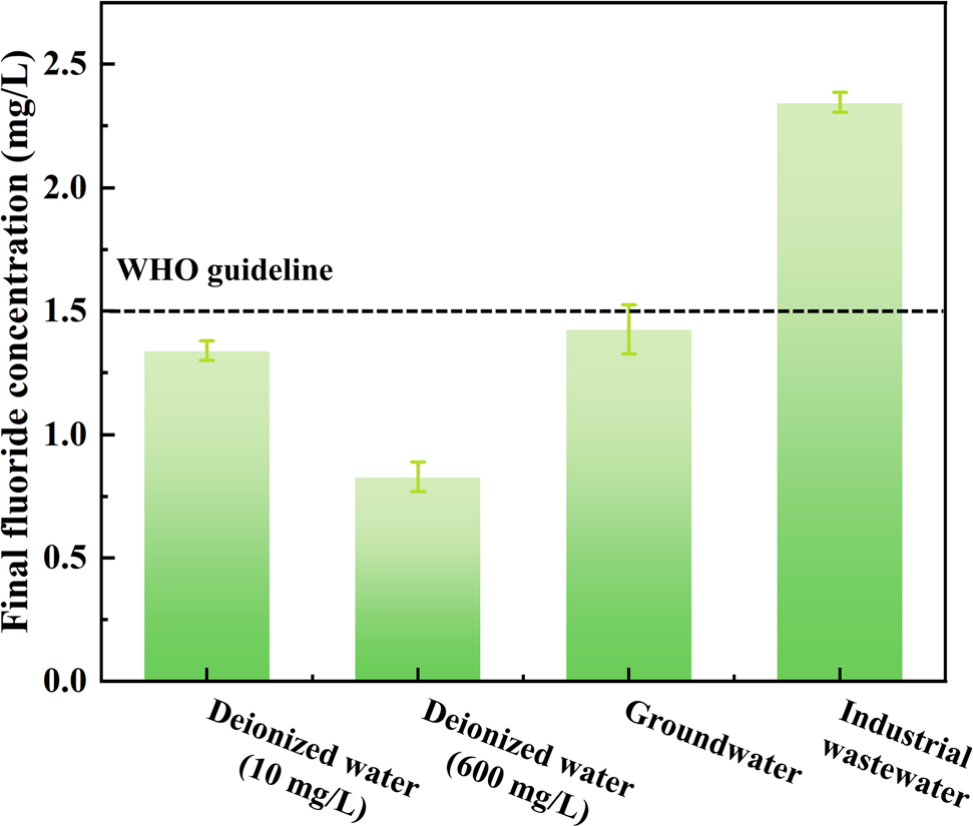
Fluoride removal from different water samples by AEG900.

To show the feasibility of AEG900 in different application environments, the fluoride removal experiments were conducted with different concentration levels of fluoride in deionized water, real groundwater, and industrial wastewater, and the results are shown in Fig. 10. According to the experimental results, we can find that when the dosage of AEG900 is 3 g L^−1^, the final concentration of fluoride can reach 0.83 mg L^−1^, meeting the guidelines of WHO (1.5 mg L^−1^). To investigate its feasibility for low fluoride concentration removal, the same dosage of AEG900 was used to treat fluoride with an initial concentration of 10 mg L^−1^, and the final fluoride is 1.43 mg L^−1^. A satisfying result can be obtained, while the low initial concentration will limit the driving force for the interaction between fluoride ions and adsorbents.

Additionally, AEG900 was applied to remove fluoride in different real water samples. The groundwater was collected in Chengdu city, China, and the industrial water was collected from the photovoltaic industry, and the detailed characterizations are listed in Table S4.† Based on the results of pre-experiments, the optimum conditions were chosen for different water samples, and the removal rates of fluoride were 99.77% for industrial wastewater and 75.54% for groundwater. It is clear that AEG900 is efficient in treating fluoride in groundwater to meet the WHO standards. For photovoltaic wastewater, lime precipitation is the most mature method for fluoride removal, but the final fluoride concentration is commonly in the range of 20 to 100 mg L^−1^, which is higher than the emission standard (15 mg L^−1^).^51^ However, in this study, by *in situ* precipitation on AEG900, the lower final fluoride concentration can be achieved without a turbidity increase in wastewater.

To sum up, AEG900 is feasible for fluoride removal within a wide range of fluoride concentrations and can reduce fluoride to the permissible concentration suggested by WHO. It also can be used in different application scenarios, and by adjusting the adsorption condition, it is feasible for industrial wastewater or groundwater treatment.

### 3.5 Comparison of other adsorbents reported in the literature

**Fig. 11:**
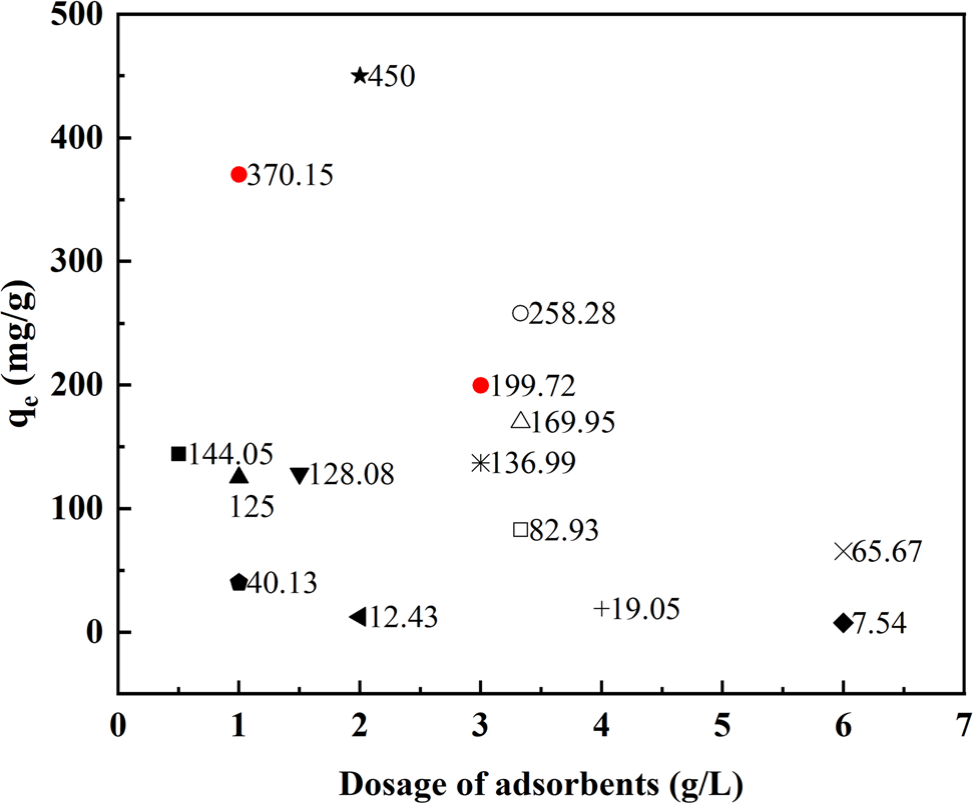
Comparation of the adsorption capacity of fluoride removal by different adsorbents with different dosages. (■: 3D porous metal oxide;^52^ □: Mytilus coruscus shells;^53^ ▲: nepheline;^44^ ▼: CaSO_4_·2H_2_O;^32^ ◆: Zr(iv)-loaded grape pomace;^54^ ○: calcined eggshells;^25^ ×: porous calcium silicate hydrates;^35^ ★: calcium hydroxide nanorods;^46^ +: charcoals;^55^ △: Sepiolite;^37^ ◀: tea powder-Zr;^56^ ◇: CeCO_3_OH nanospheres; ☆: CaO20@Al_2_O_3_;^42^●: in this study.

To confirm the superiority of AEG900, the fluoride adsorption experiment was conducted under the same condition as Lee's study.^25^ The adsorption capacity is 56.84 mg g^−1^ for AEG900 (F^−^]_0_ = 200 mg L^−1^ and [adsorbent] = 3.33 g L^−1^), which is nearly 3 times that of eggshells treated under 800 °C for 4 h as Lee *et al.* reported (18.54 mg g^−1^). Moreover, the equilibrium adsorption capacities of fluoride by different adsorbents in the literature are plotted in Fig. 11. To make the comparison meaningful, the adsorption capacities were compared with different dosages of adsorbents and the initial concentration of fluoride higher than 100 mg L^−1^ was discussed in these studies. Obviously, AEG900 owns outstanding adsorption capacity for fluoride removal, especially compared with other absorbents recovered from waste. For example, under similar conditions, the adsorption capacity of AEG900 (370.15 mg g^−1^) was nearly three times that of nepheline prepared from kaolinite (125.0 mg g^−1^).^44^ Moreover, compared with other calcium-based materials, AEG900 also shows higher adsorption performance. For example, the adsorption capacity of CaSO_4_·2H_2_O was 128.08 mg g^−1^ with 1.5 g L^−1^ dosage, which is even less than the adsorption capacity of AEG900 for fluoride with 3 g L^−1^ dosage.^32^ Besides, some synthetic adsorbents displayed higher adsorption performance than AEG900, such as calcium hydroxide nanorods (450 mg g^−1^). Compared with AEG900 recovered from eggshells, nano-size calcium hydroxide with higher SSA and the content of Ca(OH)_2_ is favorable for the adsorption and precipitation of fluoride in water, but meanwhile, the higher preparation cost and the risk of nanoparticle leaching remains.^46^ Hence, AEG900, with its low-cost, easy preparation method, and high fluoride removal efficiency, displays application potential for fluoride removal.

## Conclusion

In this study, a modified preparation method was proposed to improve fluoride removal in an aqueous solution by Ca(OH)_2_ derived from calcined eggshells. By an aging treatment in a humid environment, CaO as the main active component in calcined eggshells could convert to Ca(OH)_2_ to realize a great degree of improvement for fluoride removal, wherein the fluoride removal rate of EG900 increased nearly by 29.21%. The single-factor experiments demonstrate that AEG900 owns the characteristic of calcium-based adsorbents for fluoride removal, and the adsorption equilibrium process was well fitted with the Langmuir–Freundlich isotherm models with the maximum adsorption capacity of 370.15 mg g^−1^. Based on the adsorbent material characterization, surface complexation model analysis, and adsorption performance results, the adsorption and precipitation both contribute to the fluoride removal mechanism, in which the contribution of precipitation is bigger. Fluorite as a product is formed *in situ*, which avoids increased turbidity in the lime precipitation method. Moreover, satisfactory results of fluoride removal by AEG900 in different initial fluoride concentrations, real-life groundwater, and photovoltaic industrial wastewater were achieved, and the final fluoride could reach the requirement of WHO and the discharge standard of industrial wastewater. Overall, by aging treatment, calcined eggshells were endowed with outstanding defluorination capacity, which could be applied as a potential adsorbent for fluoride removal.

## Author contributions

Wenjing Chen: conceptualization, methodology, writing-original draft preparation, supervision. Yuanyue Wu: conceptualization, writing-review & editing. Zhiyin Xie: methodology, data curation. Yiyuan Li: writing-review & editing. Weitai Tang: formal analysis. Jinbei Yu: project administration.

## Conflicts of interest

There are no conflicts to declare.

## Supplementary Material

RA-012-D2RA05209A-s001

## References

[cit1] Gu B.-W., Lee C.-G., Park S.-J. (2018). Application of response surface methodology and semi-mechanistic model to optimize fluoride removal using crushed concrete in a fixed-bed column. Environ. Technol..

[cit2] World Health O. , Guidelines for drinking-water quality, World Health Organization, Geneva, 2011

[cit3] Vithanage M., Bhattacharya P. (2015). Fluoride in the environment: sources, distribution and defluoridation. Environ. Chem. Lett..

[cit4] EPA , National Primary Drinking Water Regulations; Announcement of the Results of EPA's Review of Existing Drinking Water Standards and Request for Public Comment and/or Information on Related Issues, 2017

[cit5] Cherukumilli K., Maurer T., Hohman J. N., Mehta Y., Gadgil A. J. (2018). Effective Remediation of Groundwater Fluoride with Inexpensively Processed Indian Bauxite. Environ. Sci. Technol..

[cit6] Jadhav S. V., Bringas E., Yadav G. D., Rathod V. K., Ortiz I., Marathe K. V. (2015). Arsenic and fluoride contaminated groundwaters: A review of current technologies for contaminants removal. J. Environ. Manage..

[cit7] Huang L., Luo Z., Huang X., Wang Y., Yan J., Liu W., Guo Y., Babu Arulmani S. R., Shao M., Zhang H. (2022). Applications of biomass-based materials to remove fluoride from wastewater: A review. Chemosphere.

[cit8] Wan K., Huang L., Yan J., Ma B., Huang X., Luo Z., Zhang H., Xiao T. (2021). Removal of fluoride from industrial wastewater by using different adsorbents: A review. Sci. Total Environ..

[cit9] Reardon E. J., Wang Y. (2000). A Limestone Reactor for Fluoride Removal from Wastewaters. Environ. Sci. Technol..

[cit10] Zhu Y. Z., Liu D. W., Liu Z. Y., Li Y. F. (2013). Impact of aluminum exposure on the immune system: A mini review. Environ. Toxicol. Pharmacol..

[cit11] Dolar D., Košutić K., Vučić B. (2011). RO/NF treatment of wastewater from fertilizer factory — removal of fluoride and phosphate. Desalination.

[cit12] EPA, The Drinking Water Treatability Database, 2010

[cit13] Millar G. J., Couperthwaite S. J., Dawes L. A., Thompson S., Spencer J. (2017). Activated alumina for the removal of fluoride ions from high alkalinity groundwater: New insights from equilibrium and column studies with multicomponent solutions. Sep. Purif. Technol..

[cit14] Alhassan S. I., Huang L., He Y., Yan L., Wu B., Wang H. (2021). Fluoride removal from water using alumina and aluminum-based composites: A comprehensive review of progress. Crit. Rev. Environ. Sci. Technol..

[cit15] George S., Pandit P., Gupta A. B. (2010). Residual aluminium in water defluoridated using activated alumina adsorption – Modeling and simulation studies. Water Res..

[cit16] Meenakshi R. C. M. (2006). Fluoride in drinking water and its removal. J. Hazard. Mater..

[cit17] Yang W., Li C., Tian S., Liu L., Liao Q. (2020). Influence of synthesis variables of a sol-gel process on the properties of mesoporous alumina and their fluoride adsorption. Mater. Chem. Phys..

[cit18] Dayananda D., Sarva V. R., Prasad S. V., Arunachalam J., Ghosh N. N. (2015). A Simple Aqueous Solution Based Chemical Methodology for Preparation of Mesoporous Alumina: Efficient Adsorbent for Defluoridation of Water. Part. Sci. Technol..

[cit19] De Angelis G., Medeghini L., Conte A. M., Mignardi S. (2017). Recycling of eggshell waste into low-cost adsorbent for Ni removal from wastewater. J. Cleaner Prod..

[cit20] Owuamanam S., Cree D. (2020). Progress of Bio-Calcium Carbonate Waste Eggshell and Seashell Fillers in Polymer Composites: A Review. J. Compos. Sci..

[cit21] Shi D., Tong H., Lv M., Luo D., Wang P., Xu X., Han Z. (2021). Optimization of hydrothermal synthesis of hydroxyapatite from chicken eggshell waste for effective adsorption of aqueous Pb(II). Environ. Sci. Pollut. Res..

[cit22] Abdel-Khalek M. A., Abdel Rahman M. K., Francis A. A. (2017). Exploring the adsorption behavior of cationic and anionic dyes on industrial waste shells of egg. J. Environ. Chem. Eng..

[cit23] Santos A. F., Arim A. L., Lopes D. V., Gando-Ferreira L. M., Quina M. J. (2019). Recovery of phosphate from aqueous solutions using calcined eggshell as an eco-friendly adsorbent. J. Environ. Manage..

[cit24] Bhaumik R., Mondal N. K., Das B., Roy P., Pal K. C., Das C., Baneerjee A., Datta J. k. (2012). Eggshell Powder as an Adsorbent for Removal of Fluoride from Aqueous Solution: Equilibrium, Kinetic and Thermodynamic Studies. E-J. Chem..

[cit25] Lee J.-I., Hong S.-H., Lee C.-G., Park S.-J. (2021). Fluoride removal by thermally treated egg shells with high adsorption capacity, low cost, and easy acquisition. Environ. Sci. Pollut. Res..

[cit26] Photiou P., Vyrides I. (2022). Calcined eggshells in anaerobic digestion: Buffering acidification in AD and evaluating end products from phosphate adsorption as soil conditioners. J. Environ. Chem. Eng..

[cit27] Jeppu G. P., Clement T. P. (2012). A modified Langmuir–Freundlich isotherm model for simulating pH-dependent adsorption effects. J. Contam. Hydrol..

[cit28] Risso R., Ferraz P., Meireles S., Fonseca I., Vital J. (2018). Highly active Cao catalysts from waste shells of egg, oyster and clam for biodiesel production. Appl. Catal., A.

[cit29] Panagiotou E., Kafa N., Koutsokeras L., Kouis P., Nikolaou P., Constantinides G., Vyrides I. (2018). Turning calcined waste egg shells and wastewater to Brushite: Phosphorus adsorption from aqua media and anaerobic sludge leach water. J. Cleaner Prod..

[cit30] Budyanto S., Kuo Y.-L., Liu J. C. (2015). Adsorption and precipitation of fluoride on calcite nanoparticles: A spectroscopic study. Sep. Purif. Technol..

[cit31] Ullah S., Al-Sehemi A. G., Mubashir M., Mukhtar A., Saqib S., Bustam M. A., Cheng C. K., Ibrahim M., Show P. L. (2021). Adsorption behavior of mercury over hydrated lime: Experimental investigation and adsorption process characteristic study. Chemosphere.

[cit32] Shao S., Ma B., Chen Y., Zhang W., Wang C. (2021). Behavior and mechanism of fluoride removal from aqueous solutions by using synthesized CaSO4·2H2O nanorods. Chem. Eng. J..

[cit33] Wei Y., Xu H., Xu S., Su H., Sun R., Huang D., Zhao L., Hu Y., Wang K., Lian X. (2020). Synthesis and characterization of calcium carbonate on three kinds of microbial cells templates. J. Cryst. Growth.

[cit34] Galván-Ruiz M., Hernández J., Baños L., Noriega-Montes J., Rodríguez-García M. E. (2009). Characterization of Calcium Carbonate, Calcium Oxide, and Calcium Hydroxide as Starting Point to the Improvement of Lime for Their Use in Construction. J. Mater. Civ. Eng..

[cit35] Guan W., Zhao X. (2016). Fluoride recovery using porous calcium silicate hydrates *via* spontaneous Ca2+ and OH− release. Sep. Purif. Technol..

[cit36] Eskikaya O., Gun M., Bouchareb R., Bilici Z., Dizge N., Ramaraj R., Balakrishnan D. (2022). Photocatalytic activity of calcined chicken eggshells for Basic Red 2 and Reactive Red 180 decolorization. Chemosphere.

[cit37] Lee J.-I., Hong S.-H., Lee C.-G., Park S.-J. (2020). Experimental and model study for fluoride removal by thermally activated sepiolite. Chemosphere.

[cit38] Sjöberg E. L., Rickard D. T. (1984). Calcite dissolution kinetics: Surface speciation and the origin of the variable pH dependence. Chem. Geol..

[cit39] Dolgaleva I. V., Gorichev I. G., Izotov A. D., Stepanov V. M. (2005). Modeling of the Effect of pH on the Calcite Dissolution Kinetics. Theor. Found. Chem. Eng..

[cit40] Moriyama S., Sasaki K., Hirajima T. (2014). Effect of calcination temperature on Mg–Al bimetallic oxides as sorbents for the removal of F− in aqueous solutions. Chemosphere.

[cit41] Zhou Q., Lin X., Li B., Luo X. (2014). Fluoride adsorption from aqueous solution by aluminum alginate particles prepared *via* electrostatic spinning device. Chem. Eng. J..

[cit42] Dayananda D., Sarva V. R., Prasad S. V., Arunachalam J., Ghosh N. N. (2014). Preparation of CaO loaded mesoporous Al2O3: Efficient adsorbent for fluoride removal from water. Chem. Eng. J..

[cit43] Saini A., Maheshwari P. H., Tripathy S. S., Waseem S., Dhakate S. R. (2020). Processing of rice straw to derive carbon with efficient de-fluoridation properties for drinking water treatment. Journal of Water Process Engineering.

[cit44] Wang H., Feng Q., Liu K., Li Z., Tang X., Li G. (2017). Highly efficient fluoride adsorption from aqueous solution by nepheline prepared from kaolinite through alkali-hydrothermal process. J. Environ. Manage..

[cit45] Nayak B., Samant A., Patel R., Misra P. K. (2017). Comprehensive Understanding of the Kinetics and Mechanism of Fluoride Removal over a Potent Nanocrystalline Hydroxyapatite Surface. ACS Omega.

[cit46] Chaudhary M., Maiti A. (2019). Defluoridation by highly efficient calcium hydroxide nanorods from synthetic and industrial wastewater. Colloids Surf., A.

[cit47] Saini A., Maheshwari P. H., Tripathy S. S., Waseem S., Gupta A., Dhakate S. R. (2021). A novel alum impregnated CaO/carbon composite for de-fluoridation of water. Groundwater for Sustainable Development.

[cit48] Turiel E., Perez-Conde C., Martin-Esteban A. (2003). Assessment of the cross-reactivity and binding sites characterisation of a propazine-imprinted polymer using the Langmuir–Freundlich isotherm. Analyst.

[cit49] El Diwani G., Amin S. K., Attia N. K., Hawash S. I. (2022). Fluoride pollutants removal from industrial wastewater. Bull. Natl. Res. Cent..

[cit50] Wang L., Di C., Li T., Chun Y., Xu Q. (2015). Preparation and catalytic behavior of biomorphic calcium oxide/carbon solid base materials. Catal. Sci. Technol..

[cit51] Palahouane B., Drouiche N., Aoudj S., Bensadok K. (2015). Cost-effective electrocoagulation process for the remediation of fluoride from pretreated photovoltaic wastewater. J. Ind. Eng. Chem..

[cit52] Wang X., Pfeiffer H., Wei J., Dan J., Wang J., Zhang J. (2022). 3D porous Ca-modified Mg-Zr mixed metal oxide for fluoride adsorption. Chem. Eng. J..

[cit53] Lee J.-I., Kang J.-K., Hong S.-H., Lee C.-G., Jeong S., Park S.-J. (2021). Thermally treated Mytilus coruscus shells for fluoride removal and their adsorption mechanism. Chemosphere.

[cit54] Zhang Y., Huang K. (2019). Grape pomace as a biosorbent for fluoride removal from groundwater. RSC Adv..

[cit55] Tchomgui-Kamga E., Ngameni E., Darchen A. (2010). Evaluation of removal efficiency of fluoride from aqueous solution using new charcoals that contain calcium compounds. J. Colloid Interface Sci..

[cit56] Cai H., Xu L., Chen G., Peng C., Ke F., Liu Z., Li D., Zhang Z., Wan X. (2016). Removal of fluoride from drinking water using modified ultrafine tea powder processed using a ball-mill. Appl. Surf. Sci..

