# Supplementary material for: Calcium hydroxide recycled from waste eggshell resources for the effective recovery of fluoride from wastewater
Source: RSC Adv. 2022 Oct 4;12(43):28264–78. doi: 10.1039/d2ra05209a (PMC9531252; doi:10.1039/d2ra05209a)
Supplement: RA-012-D2RA05209A-s001 [file RA-012-D2RA05209A-s001.pdf]

Electronic Supplementary Material (ESI) for RSC Advances.
This journal is © The Royal Society of Chemistry 2022

Calcium hydroxide recycled from waste eggshell resources for effective recovery of fluoride from wastewater

Wenjing Chen[a,*], Yuanyue Wu[a], Zhiyin Xie[a], Yiyuan Li[a], Weitai Tang[a], Jinbei Yu [a]

[a] College of Resources and Environment, Chengdu University of Information Technology, Chengdu, 610225, China

Corresponding information:

*Corresponding Author:

Wenjing Chen,

E-mail: wenjingchen88@126.com, Tel.: +86 28 85966913, Fax: +86 28 85966913,

Postal address: No.24 Xuefu Road, Shuangliu District, Chengdu, 610225, PR China

2.1 Materials

Waste eggshells were collected from the dining hall of Chengdu University of Information Technology. Sodium fluoride (NaF, purity>99%), sodium hydroxide (NaOH, purity>98%), sodium bicarbonate ($NaHCO_3$, purity>98%), sodium sulfate ($NaSO_4$, purity>98%), potassium hydrogen phosphate (K_2HPO_4, purity>98%), sodium chloride (NaCl, purity>98%) and HCl were purchased from KeLong Chemical Reagent Factory (Chengdu, China). All chemical reagents were analytical grade and used without further purification. Deionized water was used throughout the whole of work. Fluoride stock solutions were prepared by deionized water with concentration of 1 g/L and 100 mg/L and stored in the polypropylene regent bottles for different adsorption experiments.

3.1 Screening of adsorbent

Table S1 The pH change during adsorption process with different adsorbents and the weight loss of different adsorbent in adsorption equilibrium

Type of adsorbent	pH		Adsorbent mass		Weight loss	TDS/%
	Initial	Final	Initial	Final		
ES	6.66	10.51	0.03	0.0083	0.0217	72.33
ES500		10.56		0.0113	0.0187	62.33
ES600		10.53		0.0137	0.0163	54.33
ES700		10.75		0.0144	0.0156	52.00
ES800		12.01		0.0257	0.0043	14.33
AES800		11.94		0.0286	0.0014	4.67
ES900		12.12		0.027	0.003	10.00
AES900		12.04		0.0291	0.0009	3.00

3.2 Batch adsorption experiments

3.2.3 Adsorption isotherm

$$q_t = K_{id}t^{0.5} + C1 \quad \text{(S1)}$$

Where K_{id} ((mg·min$^{0.5}$)/g) is the diffusion rate constant, q_t (mg/g) is the adsorption amount of fluoride at time t (min) and C1 is the thickness of boundary layer.

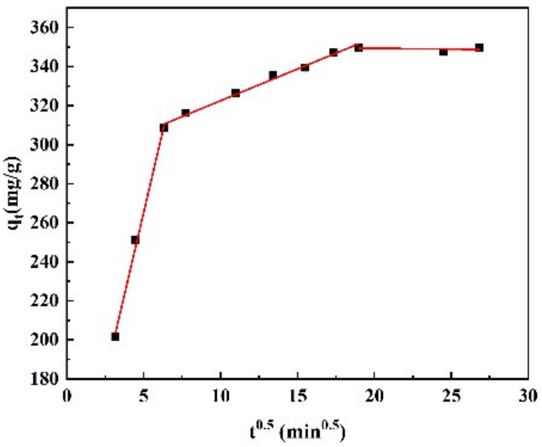

Fig. S1 The fitting results of intra-particle diffusion model

3.3 Adsorption mechanism analysis

Table S2 Input parameters for Visual MINTEQ 3.1.

Parameters	Description	Reference
Surface complexation models	Calcite-CDM (Three Plane Model)	Wolther et al 2008
Final pH	12	In this study
Solid concentration (g/L)	1	In this study
Total calcium dissolved (mg/L)	266	In this study
Specific surface area (m^2/g)	2.599	In this study
Fluoride concentration (mg/L)	5-900	In this study

Table S3 Equilibrated mass distribution of components between dissolved and precipitated phases predicted by Visual MINTEQ 3.1.

Initial fluoride (mg/L)	Total dissolved (mg/L)		% Dissolved		Total precipitated (mg/L)		% Precipitated	
	Fluoride	Calcium	Fluoride	Calcium	Fluoride	Calcium	Fluoride	Calcium
5	2.01	262.11	40.21	98.73	2.99	3.15	59.79	1.19
10	2.02	256.86	20.25	96.76	7.98	8.40	79.76	3.16
50	2.16	214.89	4.31	80.95	47.85	50.36	95.69	18.97
100	2.40	162.51	2.40	61.22	97.61	102.75	97.61	38.71
200	3.66	58.57	1.83	22.06	196.35	206.69	98.17	77.86
300	48.33	0.32	16.11	0.12	251.69	264.94	83.89	99.80
400	148.07	0.04	37.02	0.01	251.96	265.22	62.99	99.91
500	248.06	0.02	49.61	0.01	251.98	265.24	50.39	99.92
600	348.06	0.01	58.01	0.00	252.00	265.26	42.00	99.92
700	448.06	0.01	64.00	0.00	252.00	265.26	36.00	99.92
800	548.06	0.00	68.50	0.00	252.00	265.26	31.50	99.92
900	648.07	0.00	72.00	0.00	252.00	265.27	28.00	99.93

3.4 Evaluation of fluoride removal in real-life water

Table S4 Characteristics and treatment results of real industrial wastewater from photovoltaic industry and real groundwater sample.

Items	Industrial wastewater	Groundwater
Fluoride (mg/L)	1034.21	5.83
pH	1.45	6.8
DO (mg/L)	2043	4
Turbidity (NTU)	234	18
Total hardness (mg/L)	200	105
Dosage (g/L)	6	8
Equilibrium time (h)	12	24
Removal rate	99.77	75.54
